# Biosorption of Cu^2+^, Pb^2+^, Cd^2+^ and their mixture from aqueous solutions by *Michelia figo* sawdust

**DOI:** 10.1038/s41598-021-91052-2

**Published:** 2021-06-01

**Authors:** Mingzhong Long, Hong Jiang, Xiaona Li

**Affiliations:** 1 School of Geography and Ocean Science, Nanjing University, Nanjing, 210023 China; 2 College of Eco-Environmental Engineering, Guizhou Minzu University, Guiyang, 550025 China; 3 College of Pharmacy, Guizhou University of Traditional Chinese Medicine, Guiyang, 550025 China; 4 School of Karst Science, Guizhou Normal University, Guiyang, 550001 China

**Keywords:** Environmental chemistry, Biological techniques

## Abstract

The study aimed at investigating copper, lead, and cadmium removal from both single and mixed metal solutions by *Michelia figo* (Lour.) Spreng. wood sawdust treated with 0.5 mol l^−1^ NaOH for four hours. In order to evaluate the effects of each factor and interactions between factors on metal ion biosorption, a 2^3^ factorial experimental design was applied. FTIR results showed that the metal ions would bind to the hydroxyl and carboxyl groups of *M. figo* wood sawdust biomass. The main effects and interactions of three factors pH (3 and 5), initial metal ion concentration (*C*_*0*_, 0.157 and 1.574 mmol L^−1^), and dosage of biomass (*D*, 4 and 10 g L^−1^) at two levels were analyzed. The most significant variable regarding Cu^2+^ and Pb^2+^ biosorption was initial metal iron concentration. For Cd^2+^, pH was found to be the most significant factor. The maximum removal efficiencies were 94.12 and 100% for Cu^2+^ and Cd^2+^, respectively, at conditions of (+ 1, − 1, + 1): pH 5, initial metal concentration 0.157 mmol L^−1^ and dosage of biomass 10 g L^−1^, while 96.39% for Pb^2+^ at conditions of (− 1, − 1, + 1): pH 3, initial metal concentration 0.157 mmol L^−1^ and dosage of biomass 10 g L^−1^. There were some interactions between factors: pH*C_*0*_ and *C*_0_*D for Cu^2+^, pH*C_*0*_, pH*D and *C*_0_*D for Pb^2+^, pH*C_*0*_ and *C*_0_*D for Cd^2+^. Biosorption from a multi metal system showed that the presence of Cu^2+^ and Cd^2+^ had no significant influence on the Pb^2+^ removal, while Pb^2+^ in solution significantly decreased the removal efficiencies of the other two metals.

## Introduction

Metal contamination in the water environment has attracted global attention because of its severe threats to ecosystems and public health^1^. For instance, exposure to excessive levels of Pb^2+^, Cu^2+^ and Cd^2+^ significantly increases the likelihood of kidney damage, nervous system damage, and renal dysfunction as they are non-biodegradable^2^. Methods for removing heavy metals from wastewaters, such as chemical precipitation, electrochemical treatment, ion exchange, and abiological adsorption, have many disadvantages such as high cost, incomplete metal removal, and continuous input of chemicals, which makes more and more environmentalist advocate biosorption method^1^. Nonliving biomass of bacteria, fungi, algae, and waste biomass originated from organisms are all potential biosorbents^3^. As waste biomass, sawdust is a relatively abundant and inexpensive material.

Sawdust showed promising potentialities for removing environmental pollutants like dyes, oil, iodine, phenol, ammonia, and heavy metals from water^4,5^. There were some researches about chromium, copper, cadmium, nickel, and lead removal by sawdust of poplar, willow, fir, oak, maple, deodar cedar, mango tree, pine, or walnut^5–11^. Shukla concluded that both treated and untreated sawdusts were effective in the biosorption of heavy metals from water^5^.

From the 1970s to 2010s, heavy metal pollution in surface water has changed from single metal pollution to mixed metal pollution^12^. Simultaneous removal of a mixture of several heavy metals is a cost-effective method. However, compared to single metal removal, researches on multiple metal removal from solutions are much less. In a multivariate experiment, variables often correlated with each other. Employment of factorial design could test the interactions between factors and avoid the traditional one-factor-at-a-time experiments. Therefore, using a 2^3^ factorial experimental design, this work was to study the removal of copper, lead, and cadmium from aqueous single and ternary metal solutions by NaOH-treated *Michelia figo* wood sawdust. The aim was to investigate how pH, initial metal concentration, and *M. figo* sawdust biomass dosage interacted and ultimately affected copper, lead, and cadmium removal efficiencies.

## Materials and methods

### Biosorbent preparation and FTIR spectroscopy

Wood sawdust of *M. figo* was sieved to obtain particles of size range between 0.25 and 0.50 mm, and rinsed several times with deionized water. At room temperature, it was then soaked in 0.5 mol l^−1^ NaOH solution for four hours. The excess NaOH was removed by washing with deionized water. After dried at 45 °C, the biomass was stored at room temperature.

The biomass of NaOH-pretreated wood sawdust was characterized by Fourier transform infrared (FTIR) spectroscopy using FTIR spectrometer (Nicolet Nexus 870, Nicolet Instruments Co., USA). The spectrum over 4000–400 cm^−1^ was obtained with a resolution of 4 cm^−1^.

### Metal solutions

Cu^2+^, Pb^2+^ and Cd^2+^ solutions were separately prepared by diluting corresponding stock solutions (15.74 mmol l^−1^), which were obtained by dissolving analytical-reagent grade Cu(NO_3_)_2_·3H_2_O, Pb(NO_3_)_2_ and Cd(NO_3_)_2_·4H_2_O in deionized water, respectively. The mixed metal solution was prepared by diluting stock mixed solution in which the content of each metal is 5.25 mmol l^−1^. The pH was measured by pH meter and adjusted with 0.1 mol l^−1^ HNO_3_ or NaOH.

### Factorial design and batch biosorption experiments

The pH, initial concentration of metal solution, and dosage of biosorbent were employed for 2^3^ factorial design in both single and ternary metal removal experiments (Table 1). The factor levels were coded as + 1 (high level) and − 1 (low level). The statistical analyses of metal removal efficiency and removal amount were carried out using SPSS Version 13 for Windows or MINITAB Version 15 for Windows.

**Table 1:** Factors and levels used in 23 factorial design for single and ternary biosorption experiments.

Factor		Levels (coded)					
		Cu^2+^		Pb^2+^		Cd^2+^	
		− 1	+ 1	− 1	+ 1	− 1	+ 1
pH	pH	3	5	3	5	3	5
Initial metal concentration (mmol l^−1^)	*C*_0_	0.157 (0.052)^a^	1.574 (0.525)	0.157 (0.052)	1.574 (0.525)	0.157 (0.052)	1.574 (0.525)
Dosage of biomass (g l^−1^)	*D*	4	10	4	10	4	10

^a^Numbers in parenthesis represent initial metal concentration (mmol l^−1^) for ternary experiment.

The 2^3^ factorial design employed the codified regression model as follow:

1 $$\eta = A_{0} + A_{1} pH \, + A_{2} C_{0} + A_{3} D + A_{4} pHC_{0} + A_{5} pHD + A_{6} C_{0} D + A_{7} pHC_{0} D$$

where *A*_0_ represents the global mean, *A*_i_ represents the other regression coefficients, *C*_*0*_ represents initial concentration of metal solution (mmol l^−1^), and *D* represents dosage of biomass (g l^−1^).

Biosorption efficiency and amount were calculated as Eqs. (2) and (3), respectively:

2 $$\eta = \left( {\frac{C_{0} - Ce}{{C_{0}}}} \right) \times 100$$

3 $$q = \frac{{V(C_{0} - C_{e} )}}{m}$$

where *η* represents metal removal efficiency (%);*C*_*e*_ represents equilibrium concentration of metal solution (mmol l^−1^); *q* represents the amount of metal ions adsorbed on per gram of biosorbent (mmol g^−1^); *V* represents solution volume (l); and *m* represents the dry weight of sawdust biosorbent added into metal solution (g).

For each treatment, the biosorbent was added into a 250 ml Erlenmeyer flask with 100 ml of metal solution. The sorption mixture was agitated at 150 rpm for 12 h at 25 °C. In the ternary biosorption experiment, the total concentration of three species of metal ions was 0.157 (low level) or 1.574 mmol l^−1^ (high level), and each metal concentration was equal: 0.052 (low level) or 0.525 mmol l^−1^ (high level). All the experiments were performed in duplicate. After filtration and dilution, concentrations of metal solutions were analyzed using flame atomic absorption spectrometry by AA320CRT atomic absorption spectrometer (Shanghai Analytical Instrument Overall Factory, China). Standard curves were obtained respectively by examining solutions stepwise diluted of standard solutions of copper (1000ug/mL, GSBG 62,023-90), lead (1000ug/mL, GSBG 62,071-90), and cadmium (1000ug/mL, GSBG 62,040-90).

### Ethical statement

This article does not contain any studies with human participants or animals performed by any author.

### Consent for publication

This study does not contain any individual’s data.

## Results and discussion

### FTIR spectra of NaOH-treated wood sawdust

**Figure 1:**
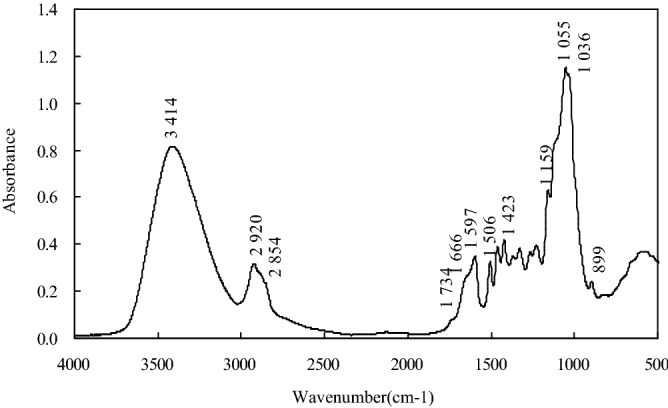
FTIR spectra of NaOH treated *Michelia figo* wood biomass.

The organic functional groups of the NaOH-treated *M. figo* sawdust and the corresponding wavenumbers were identified after comparing with other studies on infrared spectra of wood ^13,14^ or lignin^14^. Figure 1 shows the FTIR spectra of NaOH-treated *M. figo* sawdust. The bands at 3414 and 2920 cm^−1^ were assigned to O–H stretching in hydroxyl groups and C–H stretching in methyl and methylene groups, respectively. The shoulder peaks observed at 1734 and 1666 cm−^1^ were respectively considered due to the C=O bond of a carboxylic acid or its ester and C=O stretching in conjugated aryl ketone of lignin carbonyl groups. The peak at 1597 cm^−1^ was assigned to aromatic skeletal stretching plus C=O stretching. The strong peak that appeared at 1055 cm^−1^ was C–O deforming in aliphatic ethers and secondary alcohols. These results showed that the hydroxyl and carboxyl groups of NaOH-treated *M. figo* wood biomass^15^ might be the potential binding sites for the heavy metal ions.

### Results for single copper(II), lead(II) and cadmium(II) removal

Results of Cu^2+^, Pb^2+^ and Cd^2+^ removal by *M. figo* sawdust biomass are shown in Table 2. Removal results varied greatly under different experimental conditions. The maximum removal efficiencies were 94.12, 96.39 and 100% for Cu^2+^, Pb^2+^ and Cd^2+^, respectively. They were relatively higher than removal efficiencies by many other biosorbents^10,16,17^. For Cu^2+^ and Cd^2+^, the conditions at which the highest removal efficiencies occurred were pH 5, the initial metal concentration of 0.157 mmol l^−1^ and biosorbent dose of 10 g l^−1^ (+ 1, − 1, + 1), while for Pb^2+^ were pH 3, the initial metal concentration of 0.157 mmol l^−1^ and biosorbent dose of 10 g l^−1^ (− 1, − 1, + 1).

**Table 2:** Experimental factorial design results of heavy metal removal efficiency.

Run	Factor			Average removal efficiency (%)		
	pH	*C*_*0*_	*D*	Cu^2+^	Pb^2+^	Cd^2+^
1	− 1	− 1	− 1	81.19	88.44	38.17
2	− 1	− 1	+ 1	90.85	96.39	57.73
3	− 1	+ 1	− 1	27.27	31.90	28.38
4	− 1	+ 1	+ 1	58.56	64.68	43.39
5	+ 1	− 1	− 1	93.36	95.46	98.11
6	+ 1	− 1	+ 1	94.12	77.21	100.00
7	+ 1	+ 1	− 1	55.88	52.72	44.04
8	+ 1	+ 1	+ 1	94.11	86.39	76.65

At conditions of pH 5, initial metal concentration of 1.574 mmol l^−1^ and biosorbent dose of 4 g l^−1^ (+ 1, + 1, − 1), the maximum Cu^2+^, Pb^2+^ and Cd^2+^ removal amounts were 0.2151, 0.2316 and 0.1733 mmol g^−1^, respectively. NaOH-treated M. figo wood biomass presented the maximum removal amount on lead among the three species of metals. The capacity difference of biosorbent to remove bivalent Cu, Pb and Cd might be due to different adsorptive affinities of the metal ions^18^. The adsorptive affinities are tentatively correlated to cation properties, such as electronegativity^19^, hydrated radii^20^ and softness^18^.

The maximum adsorption capacities of some adsorbents reported in the literature are shown in Table 3. Compared to biomasses of algae *Ecklonia maxima* and fungus *Rhizopus arrhizus*and activated carbon, the biosorption capacity of *M. figo* sawdust treated by NaOH is relatively lower. However, it is higher than many other fungal (*Penicillium chrysogenum*), bacterial (*Enterobacter cloaceae*) and plant (Olive stone waste and *Quercus ilex*) biomasses. As a waste of timber processing, this *M. figo* sawdust is effective for removing Cu^2+^, Pb^2+^ and Cd^2+^ from aqueous solution.

**Table 3:** The maximum adsorption capacities of different adsorbents.

Biosorbents	*q*_*m*_^a^ (mmol g^−1^)			Conditions						References
	Cu^2+^	Pb^2+^	Cd^2+^	pH	*C*_*0*_ (mmol l^−1^)			*D* (g l^−1^)	T (°C)	
					Cu^2+^	Pb^2+^	Cd^2+^			
*Penicillium chrysogenum*^b^	0.14	0.56	0.10	4.5	1.22	1.22	1.22	2.00	23	Niu et al.^21^
*Rhizopus arrhizus*^b^	–	0.27	0.24	5.5	–	–	–	3.00	–	Fourest and Roux^22^
*Enterobacter cloaceae*^b^	0.11	–	0.14	–	1.57	0.00	0.89	–(inoculum)	25	Iyer et al.^23^
*Ecklonia maxima*^b^	0.95	1.05	0.55	6.0	–	–	–	20.00	20	Feng and Aldrich^24^
Activated carbon^b^	0.38	0.11	0.30	6.0	–	–	–	2.00	25	Kobya et al.^25^
Olive stone waste^b^	0.03	0.04	0.07	5.5	0.2	0.2	0.2	13.33	20 ± 2	Fiol et al.^16^
*Myriophyllum spicatum*^b^	0.16	0.23	–	< 6.0	0.16	0.05	–	20 (estimated)	25	Keskinkan et al.^26^
*Quercus ilex*^b^	0.003	0.004	0.005	6.0	0.16	0.05	0.09	10	20 ± 2	Prasad and Freitas^17^
*Pinus sylvestris* sawdust^b^	–	0.11	0.17	5.0	–	0.03	0.05	10	25	Taty-Costodes et al.^10^
*Michelia figo* sawdust^c^	0.22	0.23	0.17	5.0	1.57	1.57	1.57	4.00	25	This study

^a^The maximum adsorption capacity of biosorbent.

^b^Capacity derived from isotherm study; ^c^ estimated capacity (single metal removal experiment); T: experimental temperature.

### Statistical analysis of single metal removal efficiency

After statistical analysis of the removal efficiency results, main effects, interactions, model coefficients and associated standard errors are shown in Table 4.

**Table 4:** Statistical parameters of 2^3^ factorial design-for removal efficiency.

Factor	Species								
	Cu^2+^			Pb^2+^			Cd^2+^		
	Effect	Coefficient	Standard error	Effect	Coefficient	Standard error	Effect	Coefficient	Standard error
Average	74.42	74.42	1.27	74.15	74.15	0.93	60.81	60.81	0.80
pH	19.90	9.95	1.27	7.59	3.80	0.93	37.78	18.89	0.80
*C*_*0*_	− 30.93	− 15.46	1.27	− 30.45	− 15.22	0.93	− 25.39	− 12.69	0.80
*D*	19.98	9.99	1.27	14.04	7.02	0.93	17.27	8.63	0.80
pH * *C*_*0*_	12.18	6.09	1.27	13.67	6.84	0.93	− 13.32	− 6.66	0.80
pH * *D*	− 0.49	− 0.25	1.27	− 6.33	− 3.17	0.93	− 0.02	− 0.01	0.80
*C*_*0*_ * *D*	14.78	7.39	1.27	19.19	9.59	0.93	6.54	3.27	0.80
pH * *C*_*0*_ * *D*	3.96	1.98	1.27	6.77	3.39	0.93	8.82	4.41	0.80

Substituting the coefficients *A*_i_ in Eq. (1) with their values in Table 4, we got:

4 $$\eta_{{{\text{Cu}}^{2 + } }} = 74.42 + 9.95pH{-}15.46C_{0} + 9.99D + 6.09pHC_{0} {-}0.25pHD + 7.39C_{0} D + 1.98pHC_{0} D$$

5 $$\eta_{{{\text{Pb}}^{{2 + }} }} = 74.15 + 3.80pH{-}15.22C_{0} + 7.02D + 6.84pHC_{0} {-}3.17pHD + 9.59C_{0} D + 3.39pHC_{0} D$$

6 $$\eta_{{{\text{Cd}}^{{2 + }} }} = 60.81 + 18.89pH{-}12.69C_{0} + 8.63D{-}6.66pHC_{0} {-}0.01pHD + 3.27C_{0} D + 4.41pHC_{0} D$$

The main effects refer to deviations of the average between high and low levels for each of them. A positive effect means that, when the factor changes from low to high, there is an increase in the removal efficiency. In contrast, a negative effect means an increase in factor levels leads to decreased metal removal efficiency. For example, in the case of Cd^2+^, if a variation of pH value from 3 to 5 was made, the increase of 37.78% in the removal efficiency was observed; but for Pb^2+^, a change in initial solution concentration (*C*_*0*_) from 0.157 to 1.574 mmol l^−1^ resulted in 30.45% decrease in the metal removal efficiency.

#### Analysis of variance (ANOVA)

The sum of squares for estimating the effects and *F*-ratios of factors are presented in Table 5. Since tabulated *F*_0.05,1,8_ was equal to 5.32, all main effects and interactions with an *F* value higher than 5.32 show statistical significance. Furthermore, the effects are also statistically significant when the *P*-value is less than 0.05.

**Table 5:** Analysis of variance-full model fitting for removal efficiency.

Factor	Species								
	Cu^2+^ ^a^			Pb^2+^ ^b^			Cd^2+^ ^c^		
	Sum of squares	*F*	*P*-value	Sum of squares	*F*	*P*-value	Sum of squares	*F*	*P*-value
pH	1 583.64	61.87	0.000049	230.51	16.62	0.003552	5 708.94	562.69	0.000000
*C*_*0*_	3 826.04	149.47	0.000002	3 708.51	267.33	0.000000	2 578.36	254.13	0.000000
*D*	1 596.80	62.38	0.000048	788.35	56.83	0.000067	1 192.84	117.57	0.000005
pH * *C*_*0*_	593.41	23.18	0.001330	747.61	53.89	0.000081	709.56	69.94	0.000032
pH * *D*	0.97	0.04	0.850493	160.34	11.56	0.009364	0.00	0.00	0.992109
*C*_*0*_ * *D*	873.50	34.12	0.000386	1 472.83	106.17	0.000007	171.15	16.87	0.003404
pH * *C*_*0*_ * *D*	62.73	2.45	0.156125	183.54	13.23	0.006614	310.91	30.64	0.000550
Error	204.79			110.98			81.17		
Corrected Total	8 741.87			7 402.66			10 752.91		

^a^*R*^2^ = 0.98 (Adjusted *R*^2^ = 0.96).

^b^*R*^2^ = 0.99 (Adjusted *R*^2^ = 0.97).

^c^*R*^2^ = 0.99 (Adjusted *R*^2^ = 0.99).

For Cu^2+^, the effects of *C*_*0*_, *D* and pH factors presented high statistical significance, and the only non-significant effects were pH * *D* and pH * *C*_*0*_ * *D*. For Pb^2+^, all the effects showed the statistical significance, among which effects of *C*_*0*_ and *C*_*0*_ * *D* presented the highest significance. For Cd^2+^, effects of pH, *C*_*0*_ and *D* presented higher statistical significance, while only pH * *D* was not statistically significant.

#### Student’s *t*-test

**Figure 2:**
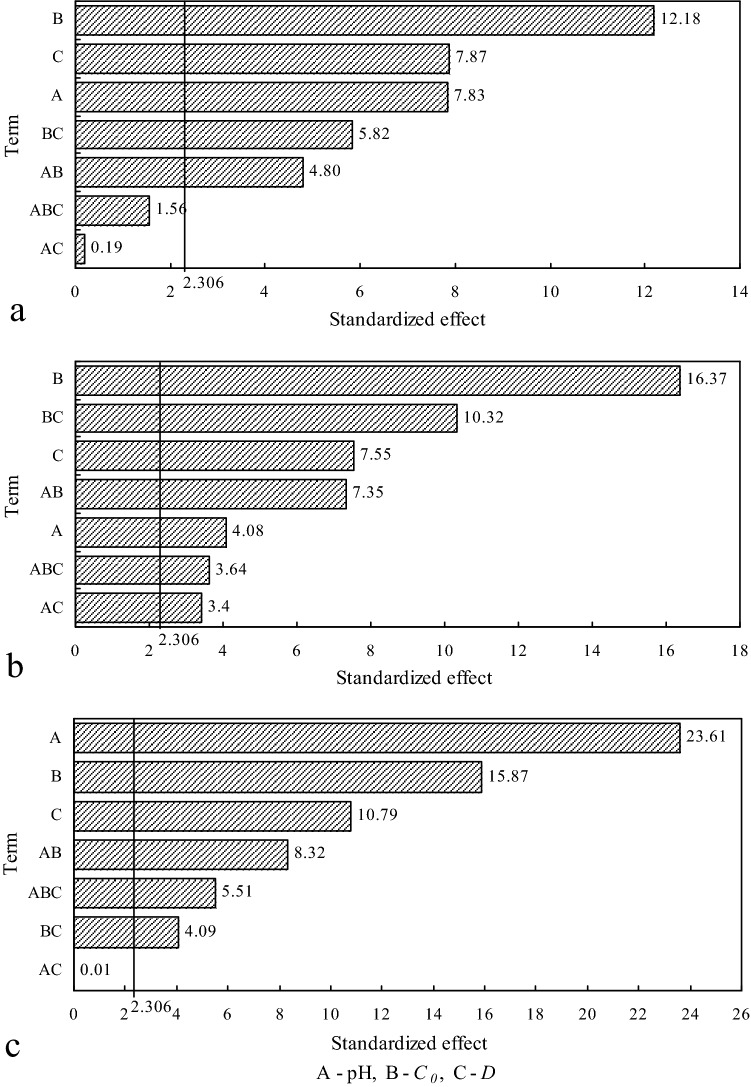
Pareto charts of effects on removal efficiency: (**a**) Cu^2+^, (**b**) Pb^2+^, (**c**) Cd^2+^.

Based on ANOVA, Student’s *t*-test was used to test whether the effects were different from zero significantly. It is showed as Pareto charts in Fig. 2, in which the vertical line indicates the minimum effect magnitude with statistical significance at 95% confidence level. All the values higher than 2.306 (*t*-value at *P* = 0.05, eight freedom degrees) were significant.

**Figure 3:**
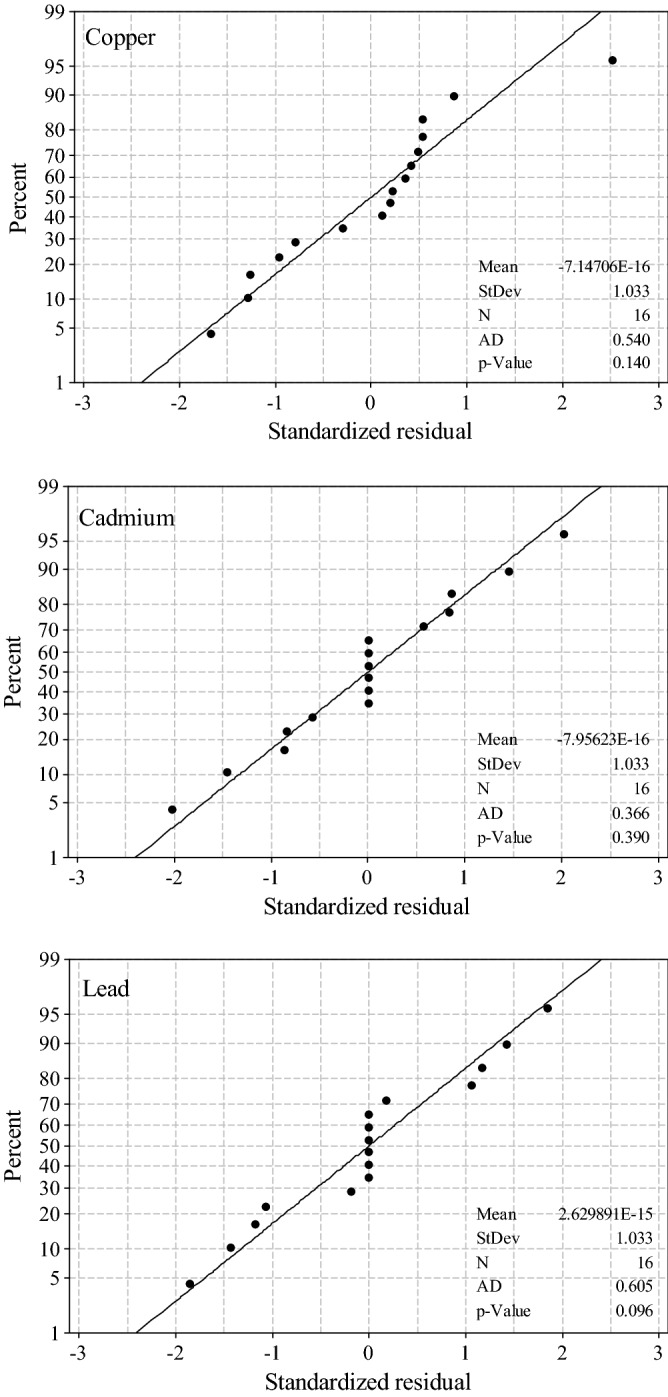
Normal probability plots of residual values for removal efficiency of Cu^2+^, Pb^2+^ and Cd^2+^.

**Figure 4:**
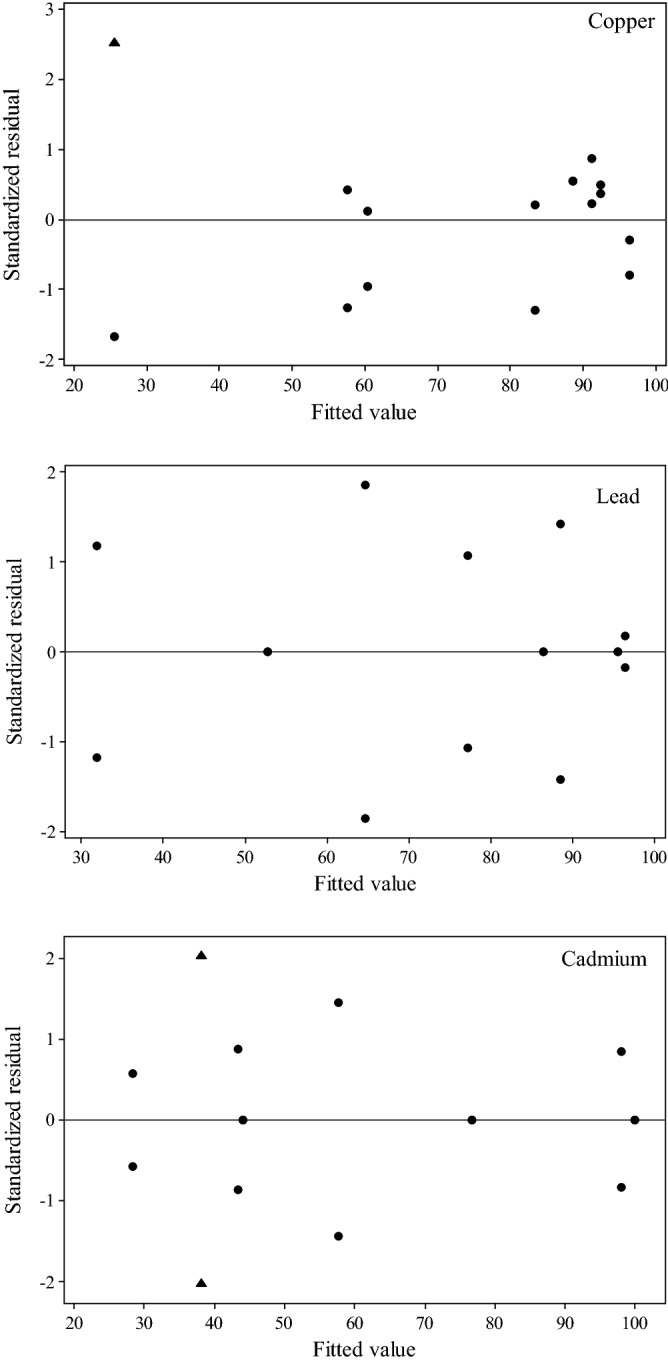
Removal efficiency for Cu^2+^, Pb^2+^ and Cd^2+^ (predicted) versus residual. Filled black triangle: outliers.

The results of the *F*-test and Student’s *t*-test suggested that the interaction effects of pH * *D* and pH * *C*_*0*_ * *D* for Cu^2+^ and pH * *D* for Cd^2+^ should be discarded. The lack of fit (Table 6) presented *F*_Cu_ = 1.24 and *F*_Cd_ = 0.00 which were much lower than tabulated *F*_0.05,2,8_ = 4.46 and *F*_0.05,1,8_ = 5.32 for Cu^2+^ and Cd^2+^, respectively. Therefore, these factors’ effects were not statistically significant. We could conclude from Fig. 3 that, in each case (Cu^2+^, Pb^2+^ or Cd^2+^), the experimental points showed a normal distribution reasonably. Figure 4 showed that the data corresponding trial 2 of run 3 for Cu^2+^ and two trials of run 1 for Cd^2+^ were considered to be outliers. After a series of statistical analyses above, it was noticed that there was no outlier point for Pb^2+^. Elimination of these points indeed reduced the lack of fit, indicating that they were really outliers.

**Table 6:** Analysis of variance-reduced models fitting for Cu^2+^ and Cd^2+^.

Factor	Statistics				
	Sum of squares	df	Mean square (MS)	*F*	*P*-value
Cu^2^^+^^a^					
Model	8 473.39	5	1 694.68	63.12	0.000
Residual error	268.48	10	26.85		
Lack of fit	63.70	2	31.85	1.24	0.338
Pure error	204.79	8	25.60		
Corrected Total	8 741.87	15			
Cd^2^^+^^b^					
Model	10 671.75	6	1 524.54	150.26	0.000
Residual error	81.17	9	9.02		
Lack of fit	0.00	1	0.00	0.00	0.992
Pure error	81.17	8	10.15		
Corrected total	10 752.91	15			

^a^*R*^2^ = 0.97 (adjusted *R*^2^ = 0.95).

^b^*R*^2^ = 0.99 (adjusted *R*^2^ = 0.99).

After further analysis of variance for Cu^2+^, Pb^2+^ and Cd^2+^, the final reduced models were:

7 $$\eta_{{{\text{Cu}}^{{2 + }} }} = 73.39 + 10.98pH{-}16.50C_{0} + 11.02D + 7.12pHC_{0} + 8.42C_{0} D$$

5 $$\eta_{{{\text{Pb}}^{{2 + }} }} = 74.15 + 3.80pH{-}15.22C_{0} + 7.02D + 6.84pHC_{0} {-}3.17pHD + 9.59C_{0} D + 3.39pHC_{0} D$$

8 $$\eta_{{{\text{Cd}}^{{2 + }} }} = 60.82 + 18.88pH{-}12.70C_{0} + 8.63D{-}6.65pHC_{0} + 3.28C_{0} D + 4.40pHC_{0} D$$

**Figure 5:**
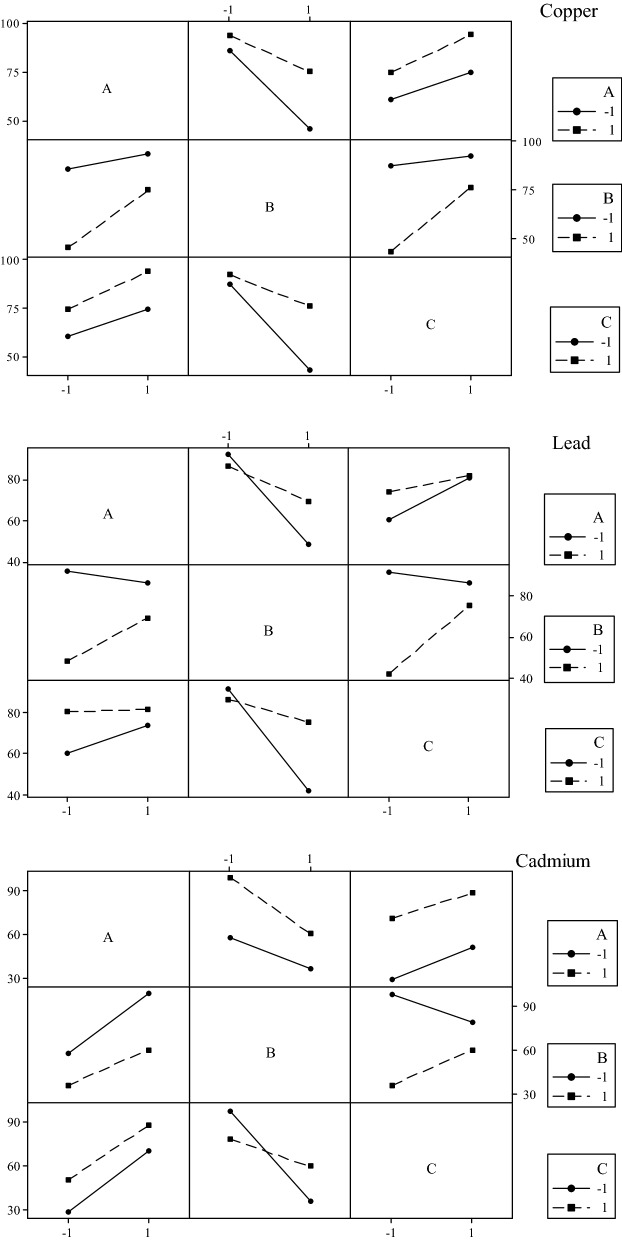
Interaction effects plot for removal efficiency of Cu^2+^, Pb^2+^ and Cd^2+^. A pH; B *C*_*0*_; C *D*.

Figure 5 illustrated the interaction effects for removal efficiency (without the outlier). It could be revealed that there were some interactions between factors, and they were pH * *C*_*0*_ and *C*_*0*_ * *D* for Cu^2+^, pH * *C*_*0*_, pH * *D* and *C*_*0*_ * *D* for Pb^2+^, pH * *C*_*0*_ and *C*_*0*_ * *D* for Cd^2+^. This result accorded with the analysis of the final reduced models.

#### Effects of factors

For all the cases (Cu^2+^, Pb^2+^ and Cd^2+^), factors pH and biosorbent dosage exhibited the same influence trend on the removal efficiencies, which was also the result of most of the biosorption works^12,27,28^. Furthermore, similar to the results of this work^29^, Ekmekyapar et al.^30^ (for Cu^2+^, biosorbent dosage lower than 5 g l^−1^ was extracted) and Amini et al.^31^ (for Cd^2+^) reported the same trend that increases in pH and biosorbent dose simultaneously with a decrease in initial metal concentration could increase the removal efficiency. Zolgharnein et al.^29^ also showed the same tendency of interaction effects pH * *C*_*0*_, pH * *D* and *C*_*0*_ * *D* with this work.

Initial metal ion concentration played the most important role in Cu^2+^ and Pb^2+^ removal. Changes in initial Cu^2+^, Pb^2+^ and Cd^2+^ concentration from 1.574 to 0.157 mmol l^−1^ resulted in 32.99, 30.45 and 25.40% increases in the removal efficiency, respectively. In the solution of higher metal concentration, there are more metal ions around the biosorbent’s active sites where metal ions would be adsorbed more sufficiently^32^. However, in this work, removal efficiency decreased at higher initial concentration might due to saturation of all functional groups.

Because solution pH impacts both biosorbates’ chemical properties and biosorbents’ surface characteristics, it is an essential factor of heavy metal removal^33^. It was found that higher unprecipitated pH is more available to the adsorption of heavy metals^34,35^. Similarly, in this study, the increase in pH value from 3 to 5 resulted in the increase of removal efficiency by 37.76, 21.96 and 7.59% for Cd^2+^, Cu^2+^ and Pb^2+^, respectively.

When the dosage of biosorbent increased from 4 to 10 g l^−1^, the removal efficiencies of Cu^2+^, Cd^2+^ and Pb^2+^ increased 22.04, 17.25 and 14.04%, respectively. That was because the increase in biosorbent dosage actually increased the adsorption sites available for binding heavy metal ions.

The interaction effect means the combined effect of factors is greater or less than expected for the straight sum of the main effects^29^. From the interaction plot (Fig. 5), respectively for Cu^2+^, Pb^2+^ and Cd^2+^, when initial metal concentration varied from 0.157 to 1.574 mmol l^−1^, removal efficiencies decreased 16.15, 11.26 and 18.85% at 10 g l^−1^ dose of NaOH-treated wood biomass, and 43.80, 49.64 and 61.90% at 4 g l^−1^. That was why, in each case, the effect of initial metal concentration was high when the biosorbent dose was low, but was lower at a higher dose. Similarly, at the lower pH 3, removal efficiencies decreased 40.75, 44.12 and 21.85% for Cu^2+^, Pb^2+^ and Cd^2+^, respectively, when initial metal concentration increased from 0.157 to 1.574 mmol l^−1^. However, at the higher pH 5, with the same initial concentration change, removal efficiencies only decreased 18.75, 16.78 and 38.71% correspondingly. For Pb^2+^, the increase of pH (from 3 to 5) resulted in 1.26 and 13.92% increase in removal efficiencies at 10 and 4 g l^−1^ biosorbent dosage, respectively.

### Ternary biosorption

**Figure 6:**
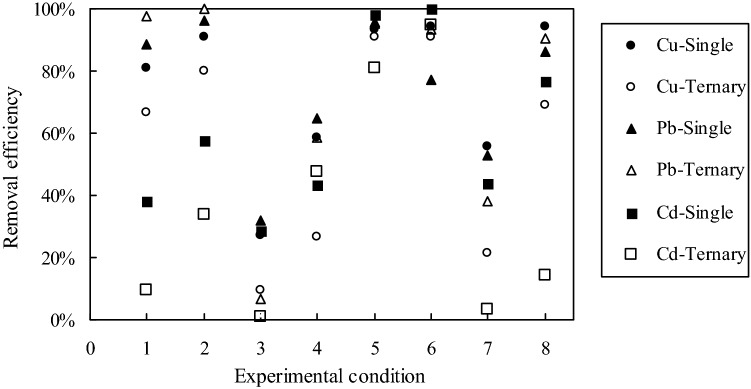
Cu^2+^, Pb^2+^ and Cd^2+^ removal efficiency from single and ternary metal solutions, Experimental condition (pH, *C*_*0*_, *D*): 1-(− 1, − 1, − 1), 2-(− 1, − 1, + 1), 3-(− 1, + 1, − 1), 4-(− 1, + 1, + 1), 5-(+ 1, − 1, − 1), 6-(+ 1, − 1, + 1), 7-(+ 1, + 1, − 1), 8-(+ 1, + 1, + 1).

The Cu^2+^ removal efficiencies between from single and ternary solutions were significantly different (*P* < 0.05), and so did Cd^2+^. However, no significant difference was obtained for Pb^2+^ (*P* = 1.000). Figure 6 shows the scatter plot of Cu^2+^, Pb^2+^ and Cd^2+^ removal efficiencies from single and ternary metal solutions. On the whole, heavy metal ions were removed most sufficiently at conditions (+ 1, − 1, − 1) and (+ 1, − 1, + 1), while most un-sufficiently at condition (− 1, + 1, − 1) for both single and mixed metal experiments (Fig. 6). There was no obvious trend in efficiencies of Pb^2+^ removal from two kinds of solutions, sometimes higher for mixed metal experiments while lower for other circumstances. The highest removal efficiency from ternary and single metal solution happened both at condition (− 1, − 1, + 1), with 100% and 96.39%, respectively. At condition (− 1, + 1, − 1), however, the biosorption efficiency sharply declined to 6.90% from mixed metal solution, compared with 31.90% removal from single solution. Except for the case of Cd^2+^ at condition (− 1, + 1, + 1), biosorptions of Cu^2+^ and Cd^2+^ from ternary metal solutions were significantly lower than those from single metal solutions. The declines of Cu^2+^ and Cd^2+^ removal efficiencies might be attributed to the greater cumulative occupancy of the binding surface of NaOH-treated sawdust biomass by Pb^2+^, which has a larger ionic radius^36^. From the above results, we found that the presence of Cu^2+^ and Cd^2+^ had no substantial influence on the Pb^2+^ removal, while the lead ions in the solution seriously decreased the removal efficiencies of the other two metal ions. This conclusion was similar to Loaëc et al.’s research on lead, cadmium and zinc uptake by exopolysaccharide^37^.

## Conclusions

Because of time, energy and cost-saving, the factorial experimental design was proved to be a good technique for investigating the biosorption of copper, cadmium and lead ions removal from aqueous solutions by NaOH-treated *M. figo* wood sawdust. The results of this work clearly showed that this biomass was effective on the removal of all the three metals both from aqueous single and ternary metal solutions. At the same conditions of pH 5, initial concentration of 1.574 mmol l^−1^ (single metal solution) and biosorbent dose of 4 g l^−1^, *M. figo* sawdust showed maximum removal amounts of 0.2151, 0.2316 and 0.1733 mmol g^−1^ for Cu^2+^, Pb^2+^ and Cd^2+^, respectively. Correspondingly, up to 94.12, 96.39 and 100.00% removal were achieved with initial single-metal-solution concentration 0.157 mmol l^−1^ and biosorbent dosage 10 g l^−1^. The most significant effect for Cu^2+^ and Pb^2+^ was ascribed to factor *C*_*0*_, while pH for Cd^2+^. Among interaction effects, pH * *C*_*0*_ and *C*_*0*_ * *D* both had reasonable influences on removing the three metals. Except for Pb^2+^, almost all the removal efficiencies of Cu^2+^ and Cd^2+^ from ternary metal solutions were significantly lower than those from single metal solutions. The presence of Cu^2+^ and Cd^2+^ had no significant influence on the Pb^2+^ removal by NaOH-treated *M. figo* wood sawdust, while the lead ions in the solution seriously decreased the removal efficiencies of the other two metals. This work concluded that NaOH-treated *M. figo* wood sawdust was cheap and effective for removing Cu^2+^, Pb^2+^ and Cd^2+^ from aqueous solution. In the future, many further researches, such as more detailed biomass characterization using multiple methods, maximum adsorption capacity modeled by adsorption isotherm, recycle potential, etc., need to be carried out to investigate if it could be widely applied on removing heavy metal ions from industrial effluents.

## Data Availability

All data and materials are fully available without restriction.
